# Research on Sapphire Deep Cavity Corrosion and Mask Selection Technology

**DOI:** 10.3390/mi12020136

**Published:** 2021-01-27

**Authors:** Yiingqi Shang, Hongquan Zhang, Yan Zhang

**Affiliations:** 1 Electronic Science and Technology, College of Electronic Engineering, Heilongjiang University, Harbin 150001, China; 1202876@s.hlju.edu.cn; 2 The 49th Research Institute, China Electronics Technology Group Corporation, Harbin 150001, China; 1202879@s.hlju.edu.cn

**Keywords:** wet etching, sapphire, mask layer

## Abstract

Aimed at the problem of the small wet etching depth in sapphire microstructure processing technology, a multilayer composite mask layer is proposed. The thickness of the mask layer is studied, combined with the corrosion rate of different materials on sapphire in the sapphire etching solution, different mask layers are selected for the corrosion test on the sapphire sheet, and then the corrosion experiment is carried out. The results show that at 250 °C, the choice is relatively high when PECVD (Plasma Enhanced Chemical Vapor Deposition) is used to make a double-layer composite film of silicon dioxide and silicon nitride. When the temperature rises to 300 °C, the selection ratio of the silicon dioxide layer grown by PECVD is much greater than that of the silicon nitride layer. Therefore, under high temperature conditions, a certain thickness of silicon dioxide can be used as a mask layer for deep cavity corrosion.

## 1. Introduction

The traditional semiconductor pressure sensor can only be used in the low temperature range; even if silicon-on-insulator (SOI) is used, it can only work below 500 °C [1,2,3,4,5,6,7,8]. After research, it is expected that the all-sapphire pressure sensor can perform a pressure measurement in a temperature environment exceeding 1500 °C. The patterning of sapphire is one of the key technologies in sensor production. Since sapphire is a very hard, corrosion-resistant crystal with a melting point of over 2000 °C, it becomes an ideal material for high temperatures and harsh environment testing.

Using sapphire as the main material of the pressure sensor requires the formation of a sapphire sensitive cavity. The manufacturing process of the sensitive cavity is divided into sapphire substrate patterning, sapphire prebonding and sapphire bonding processes. Among them, the patterning of the sapphire substrate mainly uses dry etching (ion beam etching, inductively coupled plasma) and wet etching technology to replace mechanical processing technology [9,10,11,12,13,14,15]. Dry etching is mainly a physical method that uses plasma etching technology or reactive ion etching technology to physically bombard the surface of the material exposed to the ion atmosphere, thereby removing the material and forming a structure. Dry etching has a high anisotropy and can form a very good sidewall profile, but the dry etching rate is slow, the material selection ratio is relatively poor, the substrate is easily damaged, and the cost is high. Compared with dry etching, wet etching uses liquid chemical reagents to chemically remove materials to form structures. The advantages of wet etching are that the corrosion rate is fast under a certain crystal orientation, the material selection is relatively high, the surface damage to the material is small, the cost is low, and the production efficiency is high. Conventional processes can only etch shallow sapphire grooves, and the depth is generally about 20 μm [15,16,17]. In order to better control the sensitive depth of the pressure sensor, the selection and design of the mask layer is required. During the research process, it was found that when the wavelength of the optical fiber signal needed to match the sensitive cavity with a depth of less than 10 μm, the size and depth error of the sensitive cavity were required to be less than 1 μm or even smaller, and when the SiO_2_ film made by PECVD was thin, the density was poor, which seriously affected the surface roughness of the substrate, resulting in a greatly reduced sapphire bond synthesis rate. Increasing the thickness of the SiO_2_ film will cause a large deviation in the size of the pattern window due to the characteristics of wet etching during patterning. Therefore, it is proposed to make the mask layer by superposing SiO_2_ and the metal layer. This method not only avoids the problem of a poor compactness of thin SiO_2_ but also solves the problem of a poor bonding surface roughness after sapphire wet etching.

Sapphire prebonding technology is the basis of sapphire bonding technology, and the integrity and roughness of the bonding surface are one of the important factors for sapphire prebonding. Therefore, this paper proposes to use a composite mask layer or a mask layer of a certain thickness to increase the selection ratio of the wet etching mask layer for different process conditions. While ensuring the integrity and roughness of the sapphire bonding surface, the depth of the sapphire sensitive cavity is increased, so as to realize the fabrication of the sapphire deep cavity structure.

## 2. Preparation of Sensitive Cavity Structure

### 2.1. Sapphire Etching Principle

The chemical composition of sapphire is Al_2_O_3_, and its patterning can use wet etching technology and dry etching technology. Because sapphire is a material with an extremely high hardness, dry etching is used and the rate is slow, and due to the physical reaction of ion bombardment, the surface after etching is very uneven and cannot be used for sensors based on optical signal collection. Therefore, the wet etching technique is generally used. The chemical properties of sapphire are amphipathic, so it can react with acid solutions or alkaline solutions [18,19,20,21,22,23]. After preliminary research, the acid solution used in sapphire etching is usually a mixture of phosphoric acid and sulfuric acid in a certain proportion. The reaction principle is shown in Formulas (1) to (3):

Al_2_O_3_ + H_2_O = 2AlO(OH) (1)

Al_2_O_3_ + 6H^+^ = 2Al^3+^ + 3H_2_O (2)

AlO(OH) + 3H^+^ = Al^3+^ + 2H_2_O (3)

Alkaline solution used in sapphire etching commonly consists of potassium hydroxide, sodium hydroxide and ammonia water; the main reaction equations are as below:

Al_2_O_3_ + 2OH^−^ = 2AlO_2_^−^ + H_2_O (4)

Al(OH)_3_ + OH^−^ = AlO_2_^−^ + 2H_2_O (5)

From the above reaction equations, when sapphire reacts with alkaline solution, three Al–O bonds break apart, which have higher bond energy and also need a relatively higher energy to break apart the bonds. Therefore, the chemical reaction rate is slow. However, sapphire reacts easily with acid solution, and the reaction rate is faster. In conclusion, acid solution is used in sapphire etching.

### 2.2. The Selection Ratio

The selection ratio refers to how much faster the etching rate of one material is when compared to another under the same etching or etching conditions. It is defined as the ratio of the etching rate of the material being etched to the etching rate of another material. A high selection ratio means that only the material you want to remove is removed. A high selectivity etching process does not etch the underlying material, and the protective mask layer is not etched either. High selection ratios are necessary to ensure a critical dimension and profile control in the most advanced processes. In particular, the smaller the key size, the higher the selection ratio requirement. The selection ratio SR of the material to be etched and the material of the mask layer can be calculated by the following formula:

SR = Ef/Er(6)

**Figure 1:**
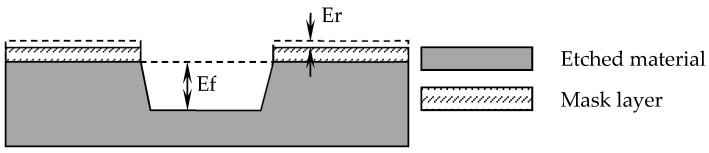
Etching selection ratio.

Among them, Ef is the etching rate of the etched material, and Er is the etching rate of the mask layer material, as shown in Figure 1.

According to this formula, the selection ratio is usually expressed as a ratio; for a process with a poor selection ratio, this ratio may be 1:1, which means that the etched material is removed as fast as the mask layer. Additionally, for a process with a high selection ratio, the ratio may be 100:1, indicating that the etching rate of the material to be etched is 100 times that of the material that does not need to be etched.

### 2.3. Sapphire Wet Etching Process

**Figure 2:**
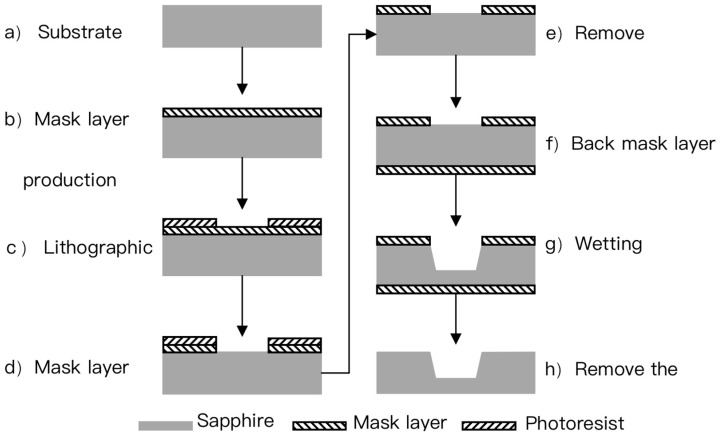
Flow chart.

A two-inch sapphire substrate is used as the substrate with a thickness of 430 μm. The main process flow is shown in Figure 2.

(a) The mask layer is made by a deposition or plating process, which serves as the mask layer for the corrosion of the sensitive cavity.
(b) The photolithography process is used to realize the patterning of the mask layer.
(c) The wet corrosion process is used to achieve the corrosion of the sensitive cavity.

According to the corrosion principle of wet corrosion, acidic solution is used as the corrosion solution, and the H+ content in phosphoric acid is relatively high, so phosphoric acid is used as the corrosion solution [2]. Sulfuric acid is added to the solution to increase the boiling point of the solution to meet the process temperature requirements. In the research process, we used three different ratios of phosphoric acid and sulfuric acid (1:1, 1:2 and 1:3) mixed solutions. When it is 1:3, the surface roughness is very small. As a photosensitive pressure sensor, the surface roughness of the sensitive film is an important indicator.

**Figure 3:**
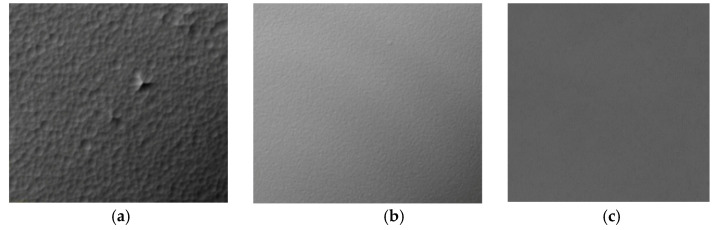
Sapphire surface states etched by different volume ratios of etching solution. (**a**) H_3_PO_4_:H_2_SO_4_ = 1:1; (**b**) H_3_PO_4_:H_2_SO_4_ = 1:2; (**c**) H_3_PO_4_:H_2_SO_4_ = 1:3.

Therefore, a mixed solution of phosphoric acid and sulfuric acid with a ratio of 1:3 is selected. The experimental results are shown in Figure 3. We set the etching time to 1 h and study the etching selection ratio of different mask layers under different etching liquid temperatures. After the etching is completed, the mask layer is removed with the corresponding etching solution to complete the sapphire etching. A three-dimensional confocal microscope was used to analyze the etched samples and calculate the etching selection ratios of different mask layers at different temperatures of the etching solution. The structure of the mask layer and the temperature of the etching solution are shown in Table 1.

To ensure the accuracy of the experiment, the mask layer structure is shown in Table 1. Temperature has a great influence on the corrosion rate of materials, which is related to the thermal motion of molecules. The higher the temperature, the faster the thermal motion of molecules. Therefore, increasing the temperature can speed up the thermal motion of solid molecules, diffuse them into the solvent and dissolve them. The speed becomes faster, and the corrosion rate increases. The selection ratio under different temperatures is studied. The thickness of the same dielectric film is the same, and the thicknesses of different dielectric films are Cr/Au: 250 nm; SiO_2_: 1 μm, SiN: 1 μm. This means that Cr/Au: 245 nm, SiO_2_/Cr/Au: 1.25 μm, SiO_2_/SiN: 2 μm, SiO_2_: 1 μm.

## 3. Results and Discussion

During the research process, four samples were made.The Cr/Au film made by the vacuum coating process is the 1# sample. After depositing the SiO2 film by PECVD, the Cr/Au film is made by the vacuum coating process, and the SiO2/Cr/Au composite mask layer is formed as the 2# sample. The SiO2/SiN composite mask layer deposited by PECVD is the 3# sample. The SiO2 mask layer deposited by PECVD is the 4# sample. After the production is completed, it is patterned, each sample is separated into four pieces, and the etching mask is studied under different temperatures.

**Figure 4:**
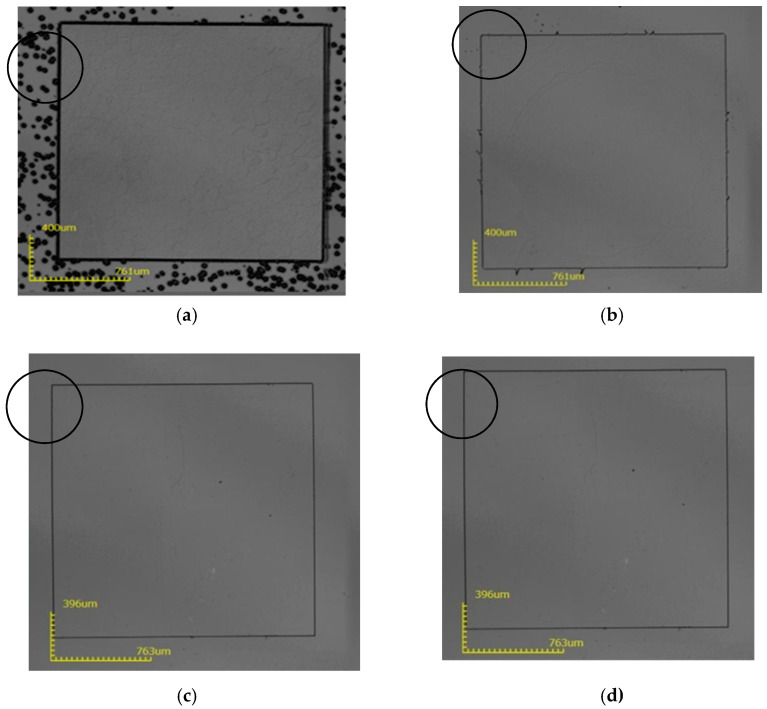
The surface state of the substrate after removing the mask layer after etching at 200 °C. (**a**) 1-1#; (**b**) 2-1#; (**c**) 3-1#; (**d**) 4-1#.

During the experiment, the thickness of the mask before and after the corrosion of the sample was tested, and the corrosion rate was calculated. The experimental results are shown in Table 2, Table 3, Table 4 and Table 5. After etching, we used a 3D confocal microscope and a surface profiler to scan the substrate, as shown in Figure 4.

It can be seen from Figure 4 that a large number of corrosion spots appeared on the surface of sample 1#, and the corrosion depth of the four samples was very small, only about 540 nm. Next, an experiment at 250 °C was performed, as seen in Table 3.

**Figure 5:**
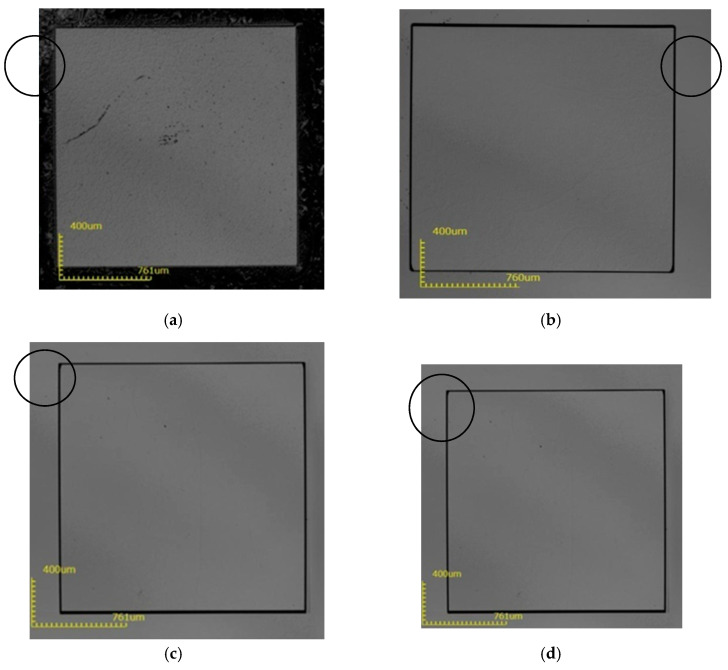
The surface state of the substrate after removing the mask layer after etching at 250 °C, (**a**) 1-2#; (**b**) 2-2#; (**c**) 3-2#; (**d**) 4-2#.

It can be seen from Figure 5 that at 250 °C, the mask area of sample 1 is completely corroded, and the other three sample mask areas are completely protected. Due to the anisotropy of the sapphire corrosion, there is a certain depth of corrosion. The side of the corrosion observed in the microscope will have a certain plane width, which is the black line in the figure. The wider the black line, the greater the corrosion depth; and the thinner the black line, the smaller the corrosion depth. At 250 °C, the corrosion depth can reach 14 μm. 

**Figure 6:**
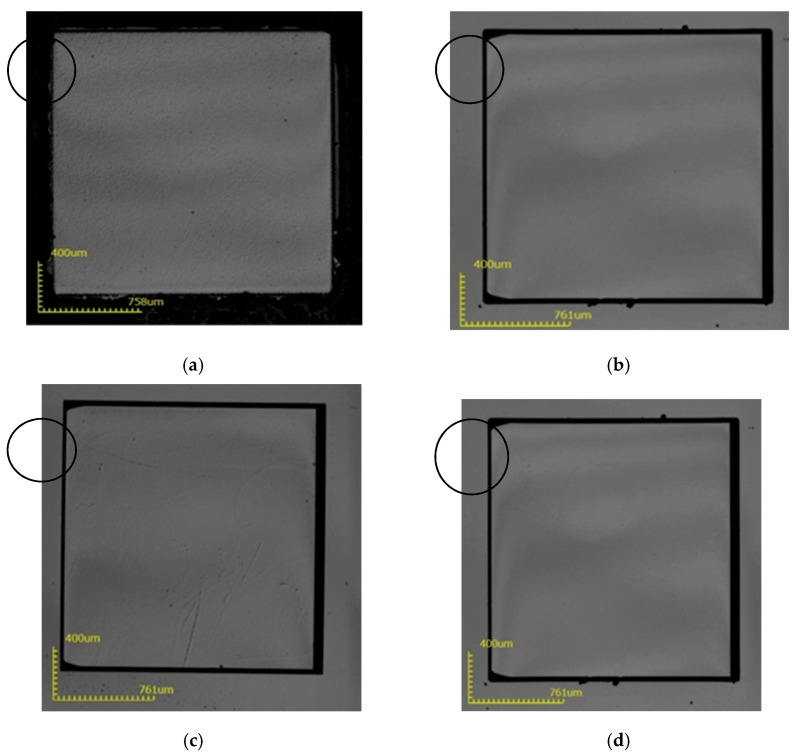
The surface state of the substrate after removing the mask layer after etching at 275 °C; (**a**) 1-3#; (**b**) 2-3#; (**c**) 3-3#; (**d**) 4-3#.

It can be seen from Figure 6 that at 275 °C, the mask area of sample 1 is completely corroded, and the other three sample mask areas are completely protected. At 275 °C, the corrosion depth can reach 49 μm, as seen in Table 4.

**Figure 7:**
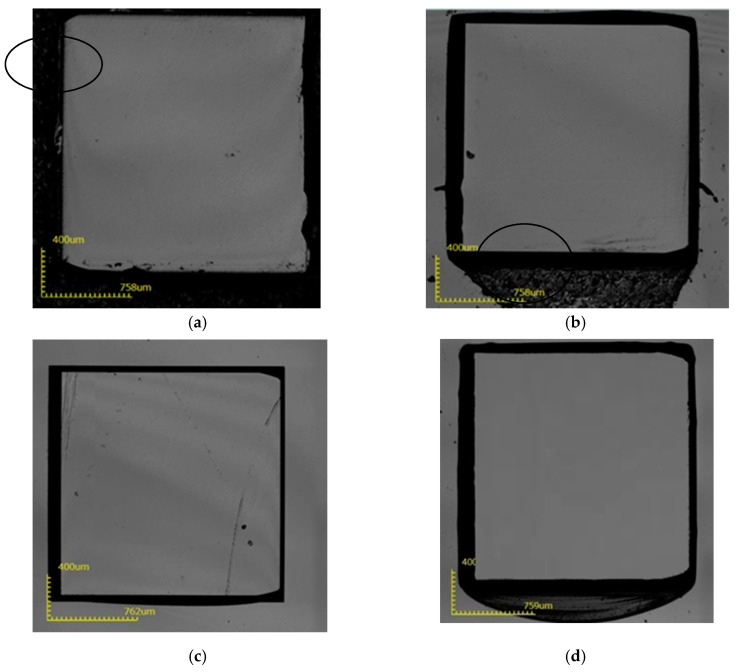
The surface state of the substrate after removing the mask layer after etching at 300 °C; (**a**) 1-4#; (**b**) 2-4#; (**c**) 3-4#; (**d**) 4-4#.

It can be seen from Figure 7 that at 300 °C, the mask area of sample 1# is completely corroded, the thickness of the mask layer of samples 2# and 4# are not enough, causing part of the mask area surface to be corroded, and the mask area of sample 3# is completely protected. At 300 °C, the corrosion depth can reach 72 μm, as seen in Table 5.

It can be concluded from the above experimental results that the wet etching of sapphire is carried out at 200–300 °C. In order to increase the adhesion of the Au layer to the sapphire surface or SiO_2_, a transition layer of Cr is added. This requires the Au layer to have good density. However, the vacuum coating method is used to prepare a Cr/Au film on a sapphire substrate. Due to the poor density and corrosion resistance of the Au layer grown, and the longer corrosion time, the corrosion solution penetrates the pores of the Au layer and enters the Cr layer, making the Cr layer be corroded and eventually causing the Cr/Au mask layer to fall off. Therefore, Cr/Au cannot be selected as the mask material. In addition, the corrosion rate at 200 °C is extremely slow, the corrosion depth of the four samples is only 540 nm, the corrosion time is long, and the depth is small, which is not suitable as a process condition. Therefore, the corrosion rates of SiO_2_, SiO_2_/SiN and sapphire are calculated according to the experimental data in Table 2 and Table 5. The results are shown in Table 6. The etching selectivity of different masks can be calculated according to Table 6, as shown in Table 7.

From the chart, one can see that the corrosion rate of SiN is significantly lower than that of SiO_2_ and sapphire at 250 °C. When the temperature increases to 275 °C, the corrosion rate of the three materials increases significantly, but the relative value of the corrosion rate of SiN is relatively large. When the temperature increases to 300 °C, the corrosion rate of SiN rapidly increases. At this time, the corrosion selection ratio of SiN/sapphire is only 3.2. It can be seen that when the temperature is lower than 250 °C, SiN/SiO_2_ can be selected as a mask material resistant to the mixed solution of H_3_PO_4_ and H_2_SO_4_. When the temperature is higher than 250 °C, SiN will not be able to be used as a mask material for the mixed solution of H_3_PO_4_ and H_2_SO_4_, and SiO_2_ material will be required to achieve a high selective ratio etching mask technology.

## 4. Conclusions

As the main sensitive unit of the optical pressure sensor, the sapphire sensitive cavity needs to receive optical fiber signals. The flatness of the sapphire etching cavity mask area is the key to the sapphire sensitive cavity bonding technology. It is very important to choose the right material to ensure the roughness of the sapphire prebonded surface. In addition, it is used to detect pressure changes under high temperature conditions and, through signal demodulation, to calculate the angle change between the reflection and refractive index of the optical signal, thereby analyzing the induced pressure changes. However, the wavelength of the optical fiber signal requires a high matching rate with the depth of the sensitive cavity, and different optical fiber signals require different sensitive cavity depths. From 10–100 μm, different depths have different application areas. The allowable error range values of the corrosion cavity depth are also different. Among them, the depth error of the sensitive cavity is an important indicator. Therefore, studying the different depths obtained by different masks is essential for pressure detection. The slower the corrosion rate, the smaller the depth, the smaller the error, the easier the size control, and the more accurate the pressure signal detection. Therefore, to make sensitive cavities of different depths for different optical fiber signals, it is very important to choose the thickness of the mask layer according to the selection ratio of the wet corrosion of materials. By studying the influence of different materials such as the thickness and combination of the mask layer on the sapphire corrosion depth in the sapphire etching solution, different mask layers are selected for experiments, and the sapphire sheet after corrosion is tested. Through experiments, the choice is relatively high when PECVD is used to make a double-layer composite film of silicon dioxide and silicon nitride at 250 °C. When the temperature rises to 300 °C, the selection ratio of the silicon dioxide layer grown by PECVD is much greater than that of the silicon nitride layer. Therefore, under high temperature conditions, a certain thickness of silicon dioxide can be used as a mask layer for deep cavity corrosion, laying the foundation for the manufacture of sapphire high-precision optical pressure sensors.

## Figures and Tables

**Table 1:** Process experiment parameters.

Scheme		Process Conditions (H_3_PO_4_:H_2_SO_4_ = 1:3)			
		Corrosive Liquid Temperature (°C)			
		200	250	275	300
Mask layer material	1	Cr/Au	/	/	/
	2	SiO_2_/Cr/Au	SiO_2_/Cr/Au	SiO_2_/Cr/Au	SiO_2_/Cr/Au
	3	SiO_2_/SiN	SiO_2_/SiN	SiO_2_/SiN	SiO_2_/SiN
	4	SiO_2_	SiO_2_	SiO_2_	SiO_2_

**Table 2:** Process results at 200 °C.

Scheme	Process Conditions (H_3_PO_4_:H_2_SO_4_ = 1:3) Corrosive Liquid Temperature (200 °C), Time (1 h)		
	Mask Thickness before Etching (nm)	Mask Thickness after Etching (nm)	Sensitive Cavity Depth (nm)
1-1#	245		
2-1#	1215	1056	540
3-1#	1966	1926	541
4-1#	999	971	540

**Table 3:** Process results at 250 °C.

Sample	Process Conditions (H_3_PO_4_:H_2_SO_4_ = 1:3) Corrosive Liquid Temperature (250 °C), Time (1 h)		
	Mask Thickness before Etching (nm)	Mask Thickness after Etching (nm)	Sensitive Cavity Depth (μm)
1-2#	245		
2-2#	1215	1024	13.8
3-2#	1966	1965	13.9
4-2#	999	990	13.9

**Table 4:** Process results at 275 °C.

Scheme	Process Conditions (H_3_PO_4_:H_2_SO_4_ = 1:3) Corrosive Liquid Temperature (275 °C), Time (1 h)		
	Mask Thickness before Etching (nm)	Mask Thickness after Etching (nm)	Sensitive Cavity Depth (μm)
1-3#	245	/	/
2-3#	1215	685	49
3-3#	1966	970	49
4-3#	999	612	49

**Table 5:** Process results at 300 °C.

Scheme	Process Conditions (H_3_PO_4_:H_2_SO_4_ = 1:3) Corrosive Liquid Temperature (300 °C), Time (1 h)		
	Mask Thickness before Etching (nm)	Mask Thickness after Etching (nm)	Sensitive Cavity Depth (μm)
1-4#	245	/	/
2-4#	1215	175	72
3-4#	1966	153	72
4-4#	999	115	72

**Table 6:** Corrosion rate of different materials.

Material	Process Conditions (H_3_PO_4_:H_2_SO_4_ = 1:3)		
	250 °C	275 °C	300 °C
Corrosion rate of SiO_2_ (nm/h)	9	387	884
Corrosion rate of SiN (nm/h)	1	1045	22,495
Corrosion rate of sapphire (μm/h)	13.8	48.8	72.3

**Table 7:** Corrosion selection ratio of different masks.

Material	Process Conditions (H_3_PO_4_:H_2_SO_4_ = 1:3)		
	250 °C	275 °C	300 °C
SiO_2_: sapphire	1533.3	126.1	81.8
SiN: sapphire	13,800.0	46.7	3.2
